# Microfluidic synthesis of alumina particle-laden beads via capillary number tuning for varying particle concentrations

**DOI:** 10.1007/s10404-026-02905-z

**Published:** 2026-06-01

**Authors:** Tao Zhang, Sebastien Jiguet, Arnaud Bertsch, Juergen Brugger

**Affiliations:** Microsystems Laboratory, Ecole Polytechnique Federale de Lausanne (EPFL), Lausanne, 1015 Switzerland

**Keywords:** Droplet microfluidics, Particle-laden inks, UV polymerization, Bead bonding

## Abstract

Colloidal particle-laden inks are increasingly used in additive manufacturing to produce functional components with tailored properties, and droplet-based microfluidics enables precise generation of high-viscosity, particle-rich droplets. Despite extensive studies on particle-free shear-thinning fluids, the coupled influence of particle concentration on both flow regimes and droplet size in microfluidic systems remains insufficiently understood. Here, we showed that particle concentration in shear-thinning, alumina-laden inks governs droplet formation dynamics through a dual effect on viscosity and interfacial tension. We investigated the droplet formation of a solvent-free, alumina-laden acrylate ink dispersed in a continuous aqueous phase using a flow-focusing microfluidic device. Inks containing 10%, 20%, and 30% (w/w) alumina particles (500 nm) were characterized in terms of rheology and interfacial tension. By adjusting flow rates of both phases and particle concentrations, we modulated the capillary number and mapped the resulting droplet formation regimes. Unlike polymer-only shear-thinning systems, where interfacial tension remains nearly constant, alumina particles modified both viscosity and interfacial tension of alumina particle-laden inks, shifting flow regimes and droplet size. Higher particle concentrations promoted jetting and altered droplet size, whereas dripping produced highly uniform droplets (< 100 μm). Downstream UV polymerization produced solid beads, and sequential exposure enabled assembly into two-dimensional structures. These results provided quantitative insight into how alumina particle concentration governs droplet generation through coupled rheological and interfacial effects, and demonstrated a bead-based assembly route for microfluidic particle-laden inks.

## Introduction

Colloidal suspensions consist of solid particles dispersed within a liquid phase and are widely used as functional inks due to their tuneable electrical, optical, mechanical, and catalytic properties. When processed via additive manufacturing techniques such as drop-on-demand inkjet printing, particle-laden inks enable the fabrication of functional components for electronics (Tan et al. 2019), energy storage (Zhao et al. 2022), photonics (Kim et al. 2009), and biomedical devices (Trombetta et al. 2017). In these processes, ink formulation plays a central role in determining printability and final material performance, with particle concentration being a key design parameter (Kiaee et al. 2021; Kim et al. 2008).

Droplet-based microfluidic printheads offer an alternative route for processing particle-laden inks, as they can generate highly uniform droplets while accommodating viscosities up to 5 Pa·s, that exceed the limits of conventional inkjet technologies (van Dijke et al. 2010). Co-flow and flow-focusing microfluidic geometries have been widely used to generate droplets from polymer solutions and particle-free shear-thinning fluids, where droplet size and flow regimes are governed primarily by viscosity, interfacial tension, and flow rate ratios (Fatehifar et al. 2021; Arratia et al. 2009; Rostami and Morini 2017).

In particle-free shear-thinning fluids, increasing polymer concentration typically raises the apparent viscosity while having little effect on interfacial tension (Shahrivar and Giudice 2021). As a result, prior studies have largely attributed changes in droplet size and breakup behaviour to viscosity-driven effects alone (Cao et al. 2024; Wang et al. 2023). However, this assumption does not necessarily extend to colloidal particle-laden inks. In such systems, particles can adsorb at fluid–fluid interfaces and modify interfacial tension, particularly at high particle loadings (Martínez-Pedrero et al. 2024; Shepherd et al. 2006).

Although particle-laden droplets and microbeads have been generated in microfluidic devices for encapsulation and material synthesis (Wang et al. 2017; Kim et al. 2010), a systematic understanding of how particle concentration simultaneously alters viscosity, interfacial tension, flow regimes, and droplet size remains limited. In particular, the combined influence of these effects on capillary number-controlled droplet generation has not been examined for particle-loaded, shear-thinning colloidal inks. Beyond droplet formation, microfluidic systems also enable in situ droplet solidification via photopolymerization or thermal curing, preserving internal particle distributions and preventing interdiffusion between neighbouring droplets (Kim et al. 2008; Parente and Sitharaman 2023). However, strategies that exploit post-solidification interfacial bonding between individual beads remain unreported, despite their potential relevance for voxel-based and modular fabrication approaches.

In this work, we investigated how alumina particle concentration (mean particle size: 500 nm) simultaneously affects viscosity and interfacial tension of shear-thinning, particle-laden inks, and how this dual effect governs droplet generation dynamics in a PDMS–glass flow-focusing microfluidic device. By varying alumina particle concentration from 10% to 30% (w/w), we examined how coupled changes in rheology and interfacial tension influence flow regime transitions and droplet size. We further demonstrated in-flow UV polymerization of droplets into solid beads and showed that sequential UV exposure enables bead–bead interfacial bonding. Together, this study provided mechanistic insight into droplet generation from particle-laden inks and highlighted a bead-based route for assembling materials.

## Results and discussion

### Rheology and interfacial tension of alumina particle-laden inks

**Fig. 1:**
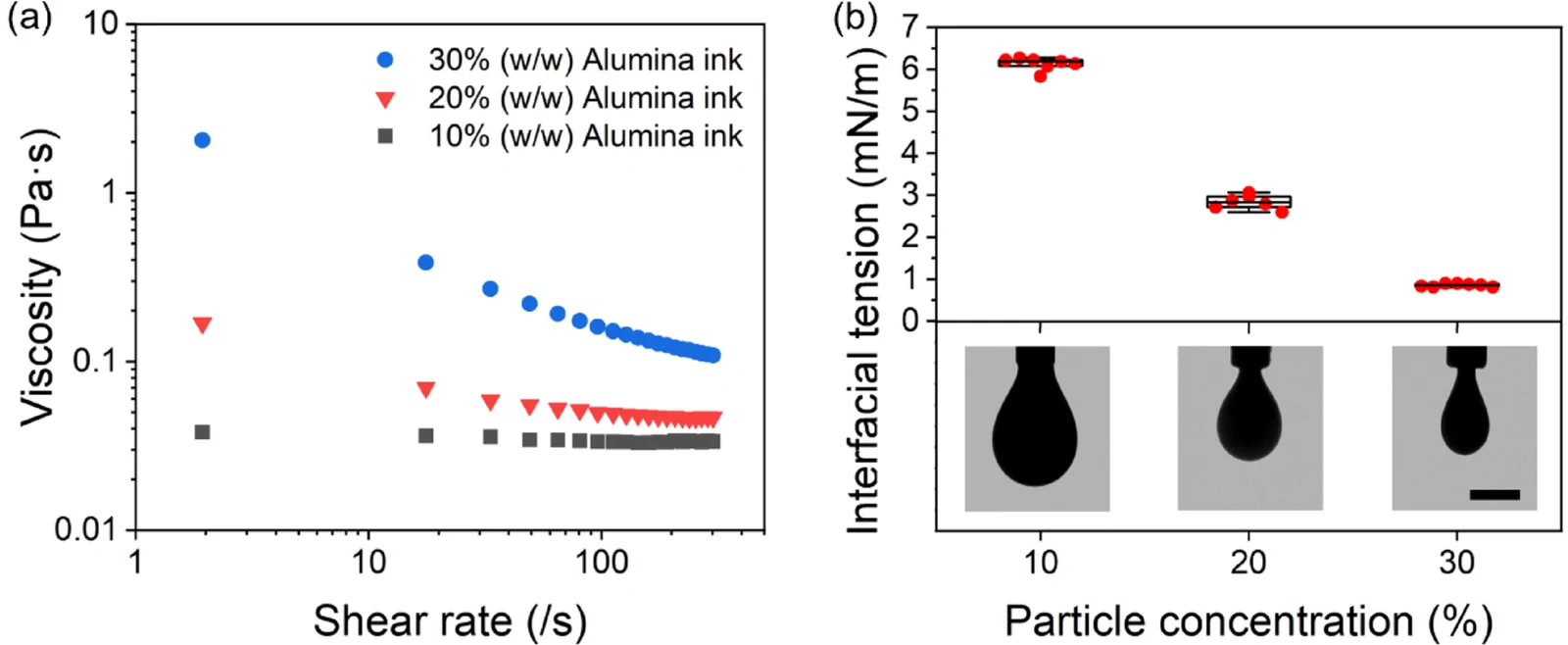
(**a**) Shear rate sweep for viscosity and shear-thinning behaviour assessment of the alumina ink formulations with three different particle concentrations, which are 10%, 20%, and 30% (w/w) of alumina particles, respectively. (**b**) Pendant drop analysis for interfacial tension measurement between the continuous phase and the alumina ink formulations with different particle concentrations. Each red dot represents a measured value. The images at the bottom show the representative pendant drops at each particle concentration; the scale bar applies to all images and represents 2 mm

At relatively low to moderate particle loadings, particle-laden inks demonstrate shear-thinning characteristics, where viscosity decreases with increasing shear rate due to the arrangement of particles along the flow direction under shear (Stickel and Powell 2005; Camargo et al. 2021). This rearrangement reduces particle entanglement, leading to a decrease in the resistance to flow. As illustrated in Fig. 1a, this shear-thinning behaviour was confirmed for alumina-laden inks composed of alumina particles dispersed in poly(ethylene glycol) diacrylate (PEGDA, Mn 250 Da), with 5% (w/w) 2,2-dimethoxy-2-phenylacetophenone (DMPA) as the photoinitiator. Increasing the particle concentration from 10% to 30% (w/w) resulted in both higher viscosity and more pronounced shear-thinning. In contrast, the continuous phase made of 4% (w/w) poly(vinyl alcohol) (PVA) dispersed in deionized water behaved as a Newtonian fluid, maintaining a constant viscosity of 5.4 mPa·s throughout the shear rate range. Furthermore, as illustrated in Fig. 1b, the interfacial tension between the alumina-laden inks and the continuous phase decreased significantly with increasing particle concentration from 10% to 30% (w/w). This reduction is attributed to the adsorption of particles at the particle-laden interface, generating a lateral surface pressure (Martínez-Pedrero et al. 2024). This pressure opposes against the direction of the interfacial tension, resulting in a lower effective interfacial tension. The observed decrease in interfacial tension is likely influenced by interactions between PVA and alumina particles. Bare alumina particles are highly hydrophilic and charged in aqueous media, which reduces their inherent tendency for interfacial adsorption. However, adsorption of PVA onto the particle surface can reduce the zeta potential (Wiśniewska et al. 2011), which would promote particle accumulation at the liquid-liquid interface. Together, these changes in viscosity and interfacial tension indicate that rising particle concentration influences droplet formation through coupled rheological and interfacial effects.

## Flow regime evolution with alumina particle concentrations

Droplet formation is primarily governed by dimensionless numbers that describe the balance of forces involved, specifically, Capillary number (Ca), Reynolds number (Re), and Weber number (We). In liquid–liquid droplet microfluidic systems, where inertial forces are negligible compared to viscous and capillary forces, the Capillary number is often used to characterize the flow (Baroud et al. 2010). It is defined as:

1 $$Ca=\frac{\mathrm{Viscous}\;\mathrm{force}}{\mathrm{Capillary}\:\mathrm{force}}=\frac{\mu\nu}{\sigma}$$

where *µ* is the apparent viscosity, *ν* is the flow velocity, and *σ* is the liquid–liquid interfacial tension between the dispersed phase and the continuous phase. The interaction between viscous and capillary forces determines the flow regimes observed during microfluidic droplet generation.

**Fig. 2:**
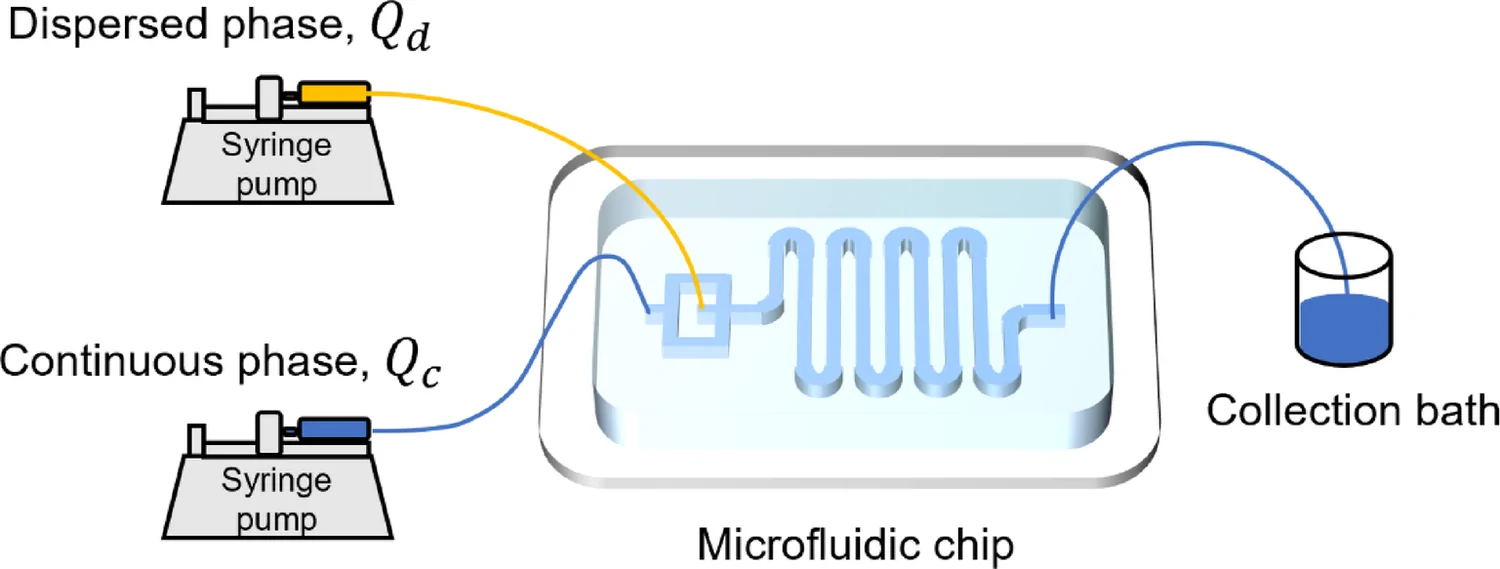
Schematic of the microfluidic experimental setup for droplet generation in a flow-focusing microfluidic chip

A flow-focusing microfluidic junction (50 μm in width, 50 μm in length, 100 μm in height) was used to investigate the droplet formation. Each experiment began by establishing a continuous liquid flow through the device to ensure the microchannel walls stayed wetted by the continuous phase. Solid particle-laden inks, serving as the dispersed phase, were then introduced into the flow-focusing junction. For flow regime mapping, the flow rate of the dispersed phase, $\:{Q}_{d}$, was kept constant while the continuous phase flow rate, $\:{Q}_{c}$, was gradually increased. The schematic of the microfluidic experimental setup is shown in Fig. 2.

**Fig. 3:**
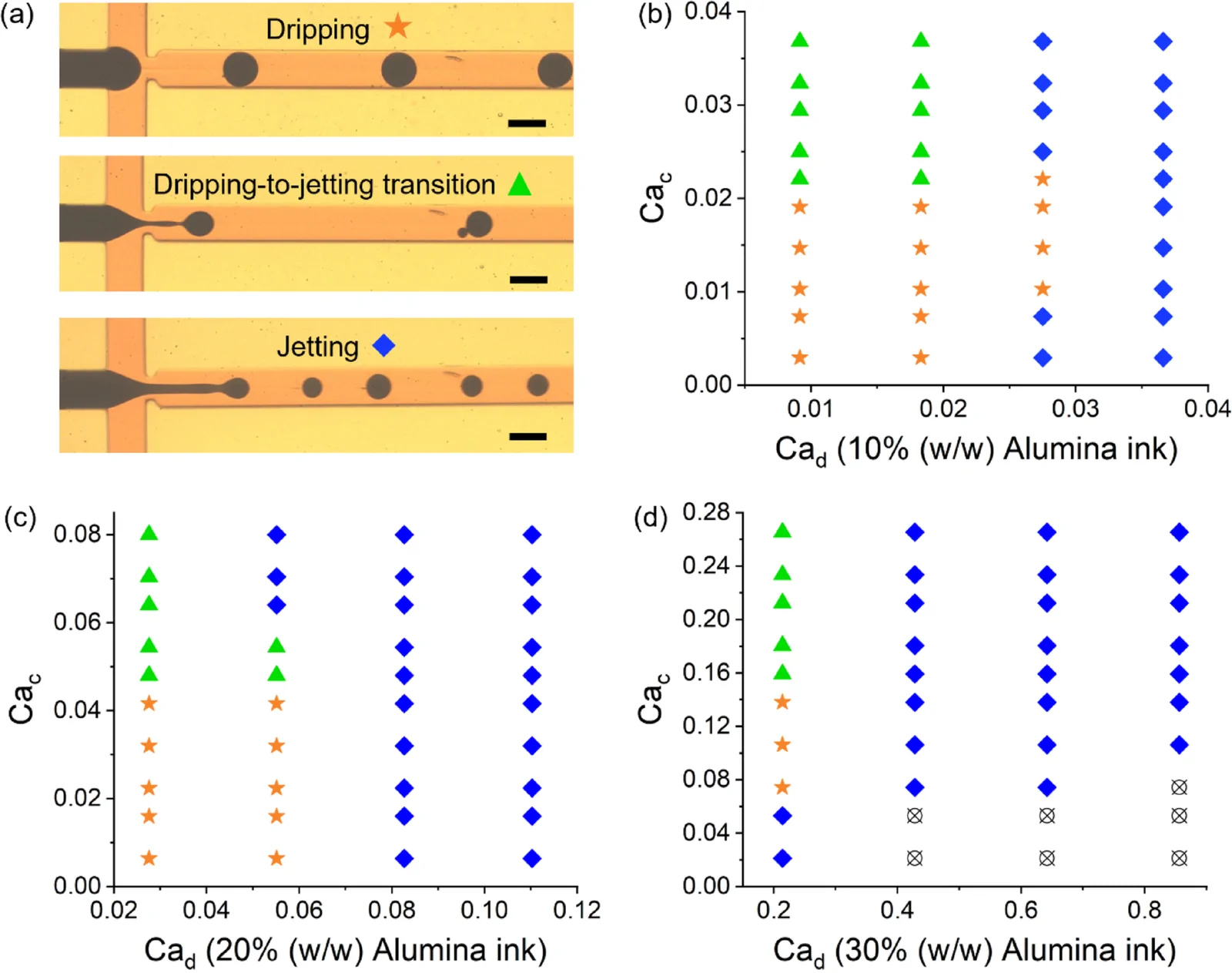
Flow regime evolution of alumina particle-laden droplet formation with varying particle concentrations in a flow-focusing microfluidic device. (**a**) Representative images of three flow regimes: Dripping, Dripping-to-Jetting Transition, and Jetting. The continuous liquid phase is shown in red due to the added red food dye while the particle-laden dispersed phase in dark grey. Scale bar: 100 μm. (**b-d**) Flow regime maps of three different concentrations of alumina inks as a function of the capillary number of the disperse phase and the continuous phase. Co-flow regime when no droplet was formed is denoted as ⊗

Over a wide range of flow conditions, we observed four distinct regimes which are defined as: Dripping, Dripping-to-jetting Transition, Jetting, and Co-flow. Figure 3a shows representative images of the flow-focusing junction illustrating each droplet generation mode. Dripping involved the formation of individual droplets that break up from the dispersed thread at the flow-focusing junction, which is preferable because uniform droplets with a narrow size distribution can be generated in this regime. The Jetting regime occurred when the dispersed phase formed a continuous jet within the main channel, breaking into droplets downstream due to Rayleigh-Plateau instability. The Transition regime is characterized by a shift in droplet formation from the junction to the end of a short jet, often producing a distinct secondary droplet following each primary one, due to breakup of a thin filament between the main droplet and the dispersed thread. The co-flow regime is defined by the absence of droplet formation near the flow-focusing junction but paralleling flows of the dispersed phase and the continuous phase.

Figures 3b–d show flow regime maps constructed using the capillary number of the continuous (Ca_c_) and dispersed (Ca_d_) phases for varying solid particle concentrations. Each regime is denoted by unique symbols and colours, i.e. Dripping (↔), Dripping-to-Jetting Transition (π), Jetting (⬥), and Co-flow (⊗). Common phenomena were seen among these three maps. Dripping generally occurred at low values of the capillary number of the dispersed phase, while increasing it, for example by increasing the flow rate of the dispersed phase, induced Jetting. Raising the capillary number of the continuous phase promoted the Transition regime before the Jetting regime. On the other hand, it can be also seen that increasing the particle concentration caused the regime to transition from the dripping to jetting. A similar regime transition phenomenon was reported in a particle-free shear thinning fluid in a microfluidic device, which was attributed to the increasing apparent viscosity with the polymer concentration (Cao et al. 2024).

## Capillary number modulation on droplet size for varying alumina particle concentrations

The dripping regime is widely used in microfluidic applications due to its ability to produce droplets with a smaller polydispersity index (PDI) compared to other regimes (Utech et al. 2015; Lashkaripour et al. 2019). In our study, we observed similar benefits: highly uniform droplets with a PDI < 5% were formed in the dripping regime while the jetting regime introduced much smaller secondary droplets alongside the primary ones, resulting in broader size variation. Therefore, we focused our analysis on the dripping regime to study how flow rate and particle concentration influence droplet size. To quantify droplet size, we defined a dimensionless droplet diameter D*:

2 $$\:{D}^{\mathrm{*}}=\:\frac{D}{{D}_{h}}$$

Where * D* and $\:{D}_{h}$ are the diameter of the droplet and the hydraulic diameter of the flow-focusing junction, respectively. For the chip used in all experiments, $\:{D}_{h}$ is 66.7 μm for the junction of 100 μm in height and 50 μm in width.

**Fig. 4:**
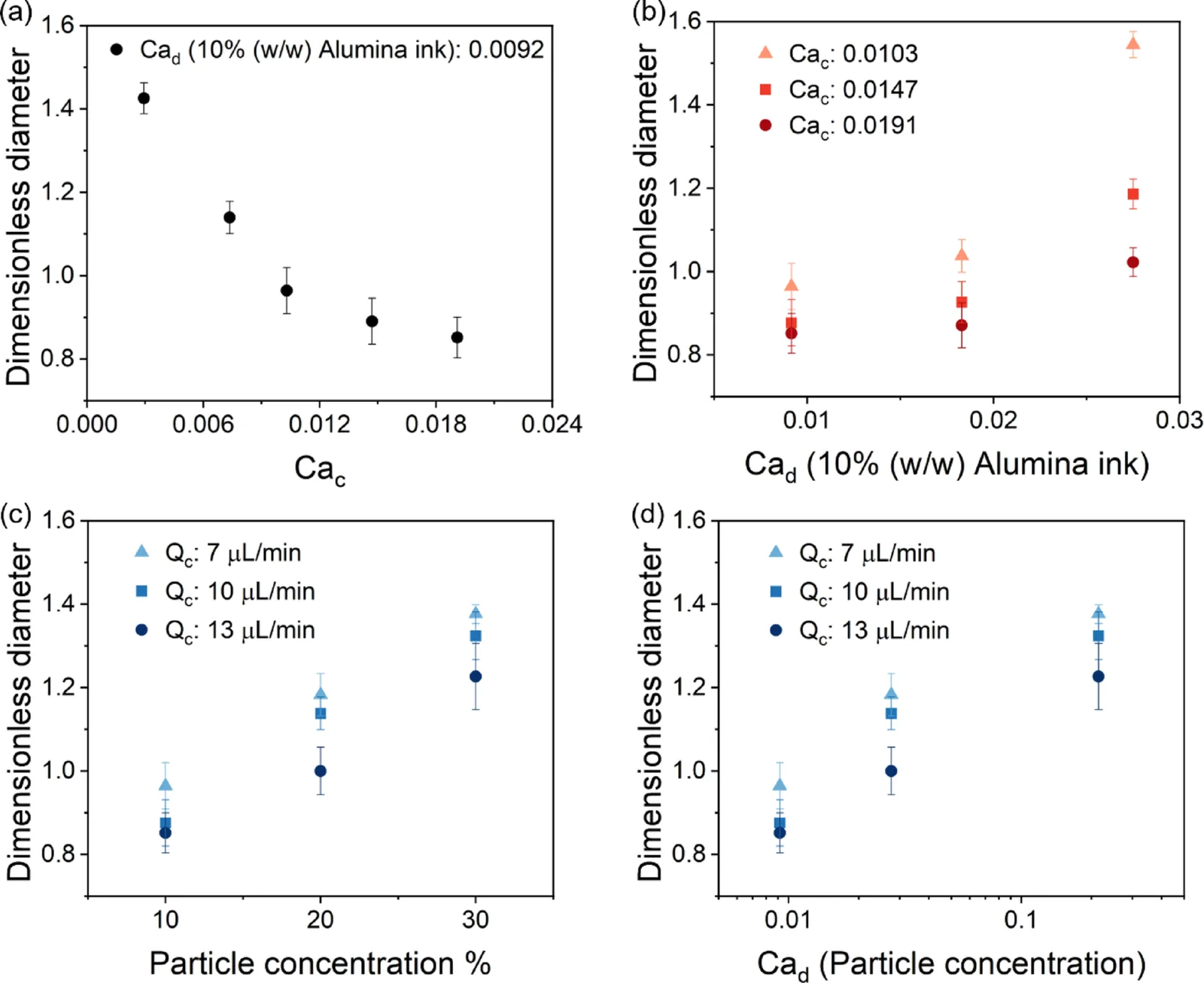
Dimensionless droplet diameter versus the capillary number and the particle concentration. (**a**) Effect of the flow rate of the continuous phase on the droplet size at a constant flow rate (i.e. Q_d_ = 1 µL/min, equivalent to Ca = 0.0092) of the 10% (w/w) alumina ink. (**b**) Effect of the flow rate of the 10% (w/w) alumina ink on the droplet size at three different flow rates of the continuous phase (i.e. Q_c_ = 7, 10, and 13 µL/min, equivalent to Ca_c_ = 0.0103, 0.0147, and 0.0191, respectively). (**c**) The droplet size was characterized with increasing the particle concentration at three different flow conditions. (**d**) Dimensionless droplet diameter as a function of the capillary number of the inks with increasing the particle concentration at three different flow conditions. Each error bar is the standard deviation of the dimensionless droplet diameter of more than 20 droplets

With 10% (w/w) alumina ink as the dispersed phase, increasing the continuous phase flow rate (while keeping the dispersed phase flow rate constant) enhanced the viscous force relative to interfacial tension, causing faster droplet breakup and reducing droplet size (Fig. 4a). Droplets with PDI < 5% were formed for low values of Ca_c_, while the size distribution went larger with increasing Ca_c_. Figure 4b shows how increasing the dispersed phase flow rate (while holding the continuous phase flow rate constant) results in larger droplets. This effect was more pronounced at lower continuous phase flow rates, where reduced shear led to increased apparent viscosity due to the shear-thinning nature of the prepared alumina inks.

To evaluate the influence of particle concentration, alumina loading was increased up to 30% (w/w). Beyond this concentration, particle clogging was observed at the dispersed-phase inlet, consistent with previous findings in a PDMS microfluidic system (Wan et al. 2012). As shown in Fig. 4c, droplet size exhibited a close to linear increase with particle concentration across different flow rates of the continuous phase while holding the dispersed phase flow rate at 1 µL/min. To capture the combined effects of viscosity and interfacial tension, the dimensionless droplet diameter was plotted against the capillary number of the ink in Fig. 4d. As the capillary number increased substantially, from below 0.01 at 10% loading to above 0.2 at 30%, the trend in droplet size became nonlinear. This result confirms that both increasing viscosity and decreasing interfacial tension contribute to elevating the capillary number and, consequently, enlarging the droplet size. These findings provide a general guideline for predicting droplet behaviour in microfluidic systems when varying particle concentrations.

## UV-induced solidification and inter-bead bonding

**Fig. 5:**
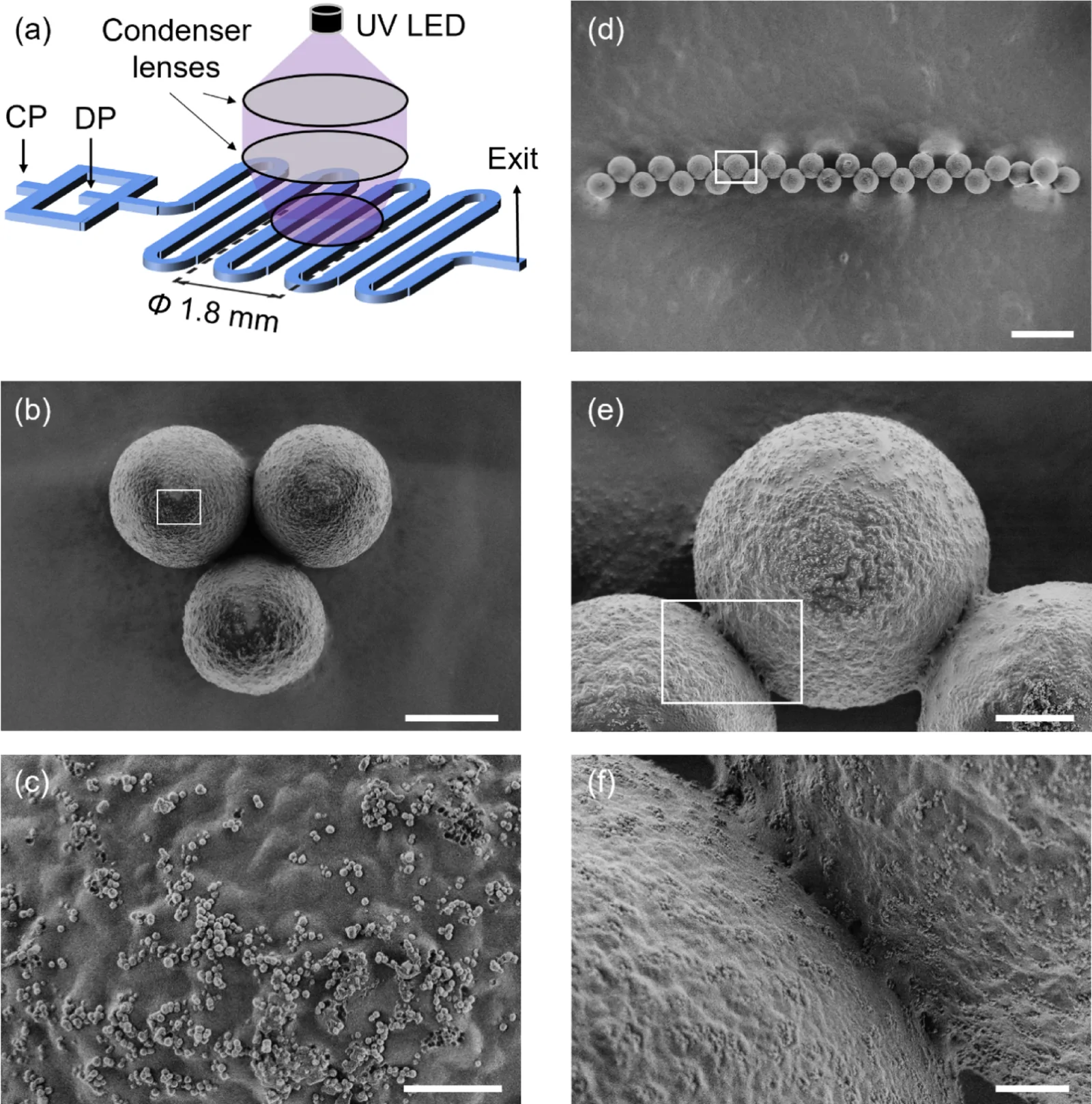
In-flow UV polymerization of droplets into solid beads. (**a**) Schematic of a flow-focusing microfluidic junction followed by a serpentine channel exposed to UV light for polymerization. CP: continuous phase, DP: dispersed phase. (**b**) SEM image showing three closely packed beads, produced from 20% (w/w) alumina ink, adhered to carbon tape. (**c**) Higher-magnification view of the white rectangle in (**b**), highlighting surface morphology of the bead. (d) SEM image of interconnected beads, also made from 20% (w/w) alumina ink, arranged in a zigzag pattern on carbon tape. (**e**) Higher-magnification view of the white rectangle in (**d**). (**f**) Closer view at the interface of beads in the white rectangle in (**e**). Scale bars: (**b**) 50 μm, (**c**) 5 μm, (**d**) 200 μm, (**e**) 20 μm, (**f**) 5 μm

Microdroplets generated in the dripping regime were polymerized in situ into solid microbeads by exposure to UV light in the downstream channel. A serpentine channel 100 μm in width was provided to increase the retention time of droplets under UV irradiation, as illustrated in Fig. 5a. The exposure time depended on the total flow rate $\:{Q}_{t}$ (equivalent to $\:{Q}_{c}+{Q}_{d}$), since the UV exposure zone which was defined by the spot size of condenser lenses had a diameter of 1.8 mm. At a total flow rate of 8 µL/min, droplets spent approximately 300 ms in this zone. Using a UV power density of 2.2 W/cm² at 365 nm, droplets were solidified into beads in flow. As shown in Fig. 5b, the resulting individual beads retained their spherical shape after polymerization. Some alumina particles detached during washing prior to scanning electron microscope (SEM), leaving voids on the bead surfaces (Fig. 5c), suggesting that these alumina particles were only physically anchored on the polymer matrix rather than chemically bound.

This method enabled the continuous and uniform production of microbeads. However, when beads accumulated in the serpentine channel, clogging occasionally occurred, rendering the device unusable. These blockages often could not be cleared by increasing the continuous-phase flow and were instead resolved by detaching the PDMS channel from the glass substrate. Bead clogging was significantly reduced when the droplet diameter was kept below 80% of the channel height, ensuring sufficient space for the continuous phase to surround the droplets during in-flow polymerization.

Bead-bead assembly was achieved in our system. As shown in Fig. 5d, a zigzag structure of inter-connected beads appeared a line direction parallel to the channel length. Notably, the beads appeared identical in size and shape, indicating that they were UV-polymerized individually before coming into contact. Figure 5e and f show that the adjacent beads were interfacially fused upon subsequent UV exposure. This interfacial fusion was made possible by the presence of a “glue-like” acrylate layer on the bead surfaces, a result of oxygen inhibition during the initial UV polymerization step. The uncured shell thickness can be tuned by the ink composition and UV light intensity (Debroy et al. 2018). While oxygen inhibition is generally suppressed in photopolymerizable resins through various strategies (Ligon et al. 2014; Bagheri and Jin 2019), here it played a constructive role in the bead assembly. The partially cured surface acted as an adhesive, enabling the beads to fuse upon subsequent UV exposure. This finding suggests a new strategy for voxelated 3D printing based on bead-by-bead assembly. It offers the intrinsic advantage of minimal material intermixing between voxels due to solidification prior to assembly. Compared to conventional droplet-based printing, this approach may offer greater flexibility in fabricating hollow and reconfigurable structures with precise control of material properties at the single-bead level.

## Conclusion

In this study, we demonstrated that alumina particle concentration influences droplet generation dynamics by simultaneously changing viscosity and interfacial tension of alumina particle-laden inks. As alumina loading increased from 10%, 20%, to 30% (w/w), the flow regime map evolved, with jetting becoming increasingly dominant and the dripping regime restricted to lower dispersed-phase flow rates. Droplet size increased with particle concentration, reflecting the combined effects of increased viscosity and reduced interfacial tension, which together elevated the capillary number of the dispersed phase. These trends were consistent across a wide range of flow conditions, highlighting the importance of considering both rheological and interfacial contributions when designing particle-laden inks for microfluidic droplet generation. By incorporating a downstream serpentine channel, we enabled in-flow UV polymerization of droplets into solid beads. Sequential UV exposure further led to interfacial bonding between adjacent beads, resulting in connected bead assemblies. This bead-based approach offers an intrinsic advantage of minimal material intermixing between units, as solidification precedes assembly.

Overall, this work provides mechanistic insight into how alumina particle concentration governs droplet formation in microfluidic systems through coupled viscosity and interfacial effects. It also demonstrates a bead-based assembly strategy in a microfluidic device, which can be beneficial for voxel-based additive manufacturing of particle-laden structures.

## Methods and materials

### Preparation of the microfluidic devices

Microfluidic devices were fabricated using photolithography and soft lithography. A physical mask was made by a laser writer (VPG200, Heidelberg Instruments). A SU-8 (3050, Kayaku) photoresist layer, 100 μm thick, was spin-coated on a silicon wafer. After a pre-baking step, the photoresist was exposed to UV light (365 nm, 250 mJ/cm²) through the physical mask, followed by post-baking, development, and hard baking at 135 °C for 2 h to create a negative master mould. To facilitate PDMS release, the mould surface was treated with trimethylchlorosilane (TMCS, Sigma Aldrich) via vapor deposition under vacuum in a desiccator for 15 min. A PDMS mixture (10:1 base-to-curing-agent mass ratio, Sylgard 184) was then degassed and poured over the master mould, cured at 80 °C for 2 h, and peeled off to yield PDMS replicas. Inlet and outlet ports were punched with a 0.75 mm biopsy punch (World Precision Instruments). The PDMS replicas and glass microscope slides were treated with oxygen plasma and bonded together immediately to form sealed microfluidic chips. To maintain hydrophilicity of the channel surfaces, the completed chips were stored in deionized water.

### Preparation of fluids

The continuous phase consisted of 4% (w/w) poly(vinyl alcohol) (PVA, Mw 31–50 kg/mol, Sigma Aldrich) dissolved in deionized water, with red food dye (Déco Relief) added for visualization. The dispersed phase was a photopolymerizable resin containing solid particles. To prepare it, 5% (w/w) of the photoinitiator 2,2-dimethoxy-2-phenylacetophenone (DMPA, Sigma Aldrich) was first mixed into poly(ethylene glycol) diacrylate (PEGDA, Mn 250 Da, Sigma Aldrich) using a roller mixer for 1 h. Solid alumina particles (D_50_ = 500 nm, AA04, Sumitomo Chemical) were then incorporated into this mixture at varying weight concentrations. The alumina particles (AA04, Sumitomo Chemical) have a manufacturer-reported median diameter D_50_ of approximately 500 nm. To ensure a homogeneous slurry, the mixture underwent a sequence of processing steps: vortex (CAPP, AHN Biotechnologie) mixing for 10 s, ultrasonication (300VT, BioLogics) at an output power of 120 Watts in a DI water bath for 2 cycles of 15 s, followed by another 10 s of vortexing. Fresh samples could be prepared by re-mixing the components immediately before droplet generation.

### Droplet generation and polymerization setup

Fluids were introduced into the inlets of the microfluidic chip using PHD ULTRA™ syringe pumps (Harvard Apparatus) through PEEK tubing (1/32” OD × 0.020” ID, IDEX). The flow rates of both the dispersed and continuous phases were entered on the user interface of the syringe pumps. Droplet formation was monitored using a high-speed camera (MIRO M310, Phantom) mounted on an inverted microscope (Eclipse TE300, Nikon) equipped with a 5× LU Plan objective (Nikon). Imaging parameters such as frame rate, resolution, and exposure time were controlled via Phantom Camera Control (PCC) software. Following any change in flow rates, imaging was initiated only after observing stable droplet breakup, typically after a 30 s delay. Images of droplets flowing inside the serpentine channels were acquired for the measurement of droplet size and calculation of size distribution. Image analysis was done via an open-source Fiji software. The individual droplet in the acquired images was detected and then the droplet area was measured. At least 20 droplets were measured at each flow condition for determining the size distribution in the dripping regime. The PDI was calculated as a ratio of the standard deviation to the mean droplet diameter. To maintain the stability of the alumina particle-laden inks, the syringes were removed and the inks were remixed whenever phase separation was observed at the inlet. This remixing was performed approximately every 15 min during droplet generation, with less frequent remixing needed for inks with higher particle concentrations. For solidified bead production, droplets were polymerized while flowing through a downstream serpentine channel exposed to UV radiation from a mounted LED (M365LP1, λ = 365 nm, 1350 mW minimum). The UV intensity at the focal plane of a pair of condenser lenses (ACL5040U-A, spot size 1.8 mm) was at 2.2 W/cm² and measured using a UV optometer (E2521264, SUSS MicroTec).

### SEM imaging

Solidified beads were retrieved from the collection vial using a 10 μm mesh filter (pluriSelect) and subsequently washed with deionized (DI) water, isopropyl alcohol, and again with DI water to remove unpolymerized surface resins resulting from oxygen inhibition. After washing, the beads were dried overnight in an oven at 80 °C. The microbead samples were imaged using a Zeiss MERLIN scanning electron microscope. It was found unnecessary to apply a conductive metal coating. Instead, the beads were simply mounted onto carbon tapes and imaged at 1 kV and 50 pA.

### Rheology

Rheological measurements were performed using a rotational rheometer (RS100 Rheostress, HAAKE) fitted with a Z20 DIN rod and a matching cylinder cup. (Each sample required 8.2 mL to fill the cup, with a set gap of 4.2 mm between the rotating rod and the bottom surface.) Measurements were taken at a constant temperature of 23 °C, with shear rate gradually increasing from 1 to 300 s⁻¹. At each shear rate, shear stress was recorded and averaged over 15 data points collected during a 15-second interval. Viscosity was then calculated using the measured shear stress and shear rate values using RheoWin 3 Data Manager software.

### Pendant drop analysis

Interfacial tension was measured using an Easy Drop system (DSA20E, Krüss), which was equipped with a backlight and a high-resolution camera. Pendant drop images were taken at the point just before detachment from an injection needle with a 1.83 mm diameter, while the needle tip was submerged in a reservoir containing 4% (w/w) polyvinyl alcohol (PVA) solution. For statistical reliability, at least six drops were recorded for each ink formulation. The captured images of droplet shape were analysed using OpenDrop, an open-source software, by fitting the Young-Laplace equation to determine the interfacial tension values (Berry et al. 2015).

## Data Availability

No datasets were generated or analysed during the current study.

## References

[CR1] Arratia PE, Cramer L-A, Gollub JP, Durian DJ (2009) The Effects of Polymer Molecular Weight on Filament Thinning and Drop Breakup in Microchannels. New J Phys 11(11):115006. 10.1088/1367-2630/11/11/115006

[CR2] Bagheri A, and Jianyong Jin (2019) Photopolymerization in 3D Printing. ACS Appl Polym Mater 1(4):593–611. 10.1021/acsapm.8b00165

[CR3] Baroud CN, Gallaire F, Rémi, Dangla (2010) Dynamics of Microfluidic Droplets. *Lab on a Chip* 10 (16): 2032. 10.1039/c001191f 20559601

[CR4] Berry JD, Neeson MJ, Dagastine RR, Derek YC, Chan, Tabor RF (2015) Measurement of Surface and Interfacial Tension Using Pendant Drop Tensiometry. J Colloid Interface Sci 454(September):226–237. 10.1016/j.jcis.2015.05.012 26037272

[CR6] Cao H, Lu Y, Wu L, Zhang C, and Liang-Liang Zhang (2024) Shear-Thinning Fluid Droplets Formation in Axisymmetric Flow-Focusing Microfluidics. Ind Eng Chem Res 63(8):3766–3779. 10.1021/acs.iecr.3c03947

[CR5] de Camargo IL, Morais MM, Fortulan CA, and Marcia Cristina Branciforti (2021) A Review on the Rheological Behavior and Formulations of Ceramic Suspensions for Vat Photopolymerization. Ceram Int 47(9):11906–11921. 10.1016/j.ceramint.2021.01.031

[CR7] Debroy D, John Oakey, and, Li D (2018) Interfacially-Mediated Oxygen Inhibition for Precise and Continuous Poly(Ethylene Glycol) Diacrylate (PEGDA) Particle Fabrication. J Colloid Interface Sci 510(January):334–344. 10.1016/j.jcis.2017.09.081 28961432

[CR9] Fatehifar M, Revell A, and Masoud Jabbari (2021) Non-Newtonian Droplet Generation in a Cross-Junction Microfluidic Channel. Polymers 13(12):12. 10.3390/polym13121915 PMC8226625 34207574

[CR10] Kiaee M, Mahdi T, Maeder, and Juergen Brugger (2021) Inkjet-Printed Composites for Room-Temperature VOC Sensing: From Ink Formulation to Sensor Characterization. Adv Mater Technol 6(1):2000929. 10.1002/admt.202000929

[CR12] Kim S-H, Jeon S-J, Yi G-R, Heo C-J, Choi JH, Seung-Man Y (2008) Optofluidic Assembly of Colloidal Photonic Crystals with Controlled Sizes, Shapes, and Structures. Adv Mater 20(9):1649–1655. 10.1002/adma.200703022

[CR11] Kim J, Yeon C, Ingrosso V, Fakhfouri et al (2009) Inkjet-Printed Multicolor Arrays of Highly Luminescent Nanocrystal-Based Nanocomposites. Small 5(9):1051–1057. 10.1002/smll.200801315 19199336

[CR13] Kim S-H, Sim JY, Lim J-M, and Seung-Man Yang (2010) Magnetoresponsive Microparticles with Nanoscopic Surface Structures for Remote-Controlled Locomotion. Angew Chem Int Ed 49(22):3786–3790. 10.1002/anie.201001031 20394094

[CR14] Lashkaripour A, Rodriguez C, Ortiz L, and Douglas Densmore (2019) Performance Tuning of Microfluidic Flow-Focusing Droplet Generators. Lab Chip 19(6):1041–1053. 10.1039/C8LC01253A 30762047

[CR15] Ligon S, Clark B, Husár H, Wutzel R, Holman, and Robert Liska (2014) Strategies to Reduce Oxygen Inhibition in Photoinduced Polymerization. Chem Rev 114(1):557–589. 10.1021/cr3005197 24083614

[CR16] Martínez-Pedrero F, Carbone C, Rubio RamónG, Ortega F, Guzmán E (2024) A Critical Examination of the Physics behind the Formation of Particle-Laden Fluid Interfaces. Curr Opin Colloid Interface Sci 74(December):101868. 10.1016/j.cocis.2024.101868

[CR17] Parente M, Jack, and Balaji Sitharaman (2023) Synthesis and Characterization of Carbon Microbeads. ACS Omega 8(37):34034–34043. 10.1021/acsomega.3c05042 PMC10515371 37744801

[CR18] Rostami B, Morini GL (2017) Micro Droplets of Non-Newtonian Solutions in Silicone Oil Flow through a Hydrophobic Micro Cross-Junction. J Phys: Conf Ser 923(1):012021. 10.1088/1742-6596/923/1/012021

[CR19] Shahrivar K, and Francesco Del Giudice (2021) Controlled Viscoelastic Particle Encapsulation in Microfluidic Devices. Soft Matter 17(35):8068–8077. 10.1039/D1SM00941A 34525163

[CR20] Shepherd RF, Jacinta C, Conrad SK, Rhodes et al (2006) Microfluidic Assembly of Homogeneous and Janus Colloid-Filled Hydrogel Granules. Langmuir 22(21):8618–8622. 10.1021/la060759+ 17014093

[CR21] Sontti SG, Atta A (2017) CFD Analysis of Microfluidic Droplet Formation in Non–Newtonian Liquid. Chem Eng J 330(December):245–261. 10.1016/j.cej.2017.07.097

[CR22] Stickel JJ, Powell RL, FLUID MECHANICS AND RHEOLOGY OF DENSE SUSPENSIONS (2005). *Annual Review of Fluid Mechanics* 37 (Volume 37, 2005): 129–49. 10.1146/annurev.fluid.36.050802.122132

[CR23] Tan H, Wei J, An CK, Chua, and Tuan Tran (2019) Metallic Nanoparticle Inks for 3D Printing of Electronics. Adv Electron Mater 5(5):1800831. 10.1002/aelm.201800831

[CR24] Trombetta R, Inzana JA, Schwarz EM, Kates SL, Awad HA (2017) 3D Printing of Calcium Phosphate Ceramics for Bone Tissue Engineering and Drug Delivery. Ann Biomed Eng 45(1):23–44. 10.1007/s10439-016-1678-3 PMC5173433 27324800

[CR25] Utech S, Prodanovic R, Mao AS, Ostafe R, Mooney DJ, Weitz DA (2015) Microfluidic Generation of Monodisperse, Structurally Homogeneous Alginate Microgels for Cell Encapsulation and 3D Cell Culture. Adv Healthc Mater 4(11):1628–1633. 10.1002/adhm.201500021 PMC4529809 26039892

[CR8] van Dijke K, Kobayashi I, Schroën K, Uemura K, Nakajima M, and Remko Boom (2010) Effect of Viscosities of Dispersed and Continuous Phases in Microchannel Oil-in-Water Emulsification. Microfluid Nanofluid 9(1):77–85. 10.1007/s10404-009-0521-7

[CR26] Wan J, Shi L, Benson B et al (2012) Microfluidic Generation of Droplets with a High Loading of Nanoparticles. Langmuir 28(37). 10.1021/la3025952 PMC3856230 22934976

[CR27] Wang J, Li Y, Wang X et al (2017) Droplet Microfluidics for the Production of Microparticles and Nanoparticles. Micromachines 8(1):22. 10.3390/mi8010022

[CR28] Wang Z, Xiang X, Zhu H et al (2023) Generation of Droplets of Shear-Thinning Non-Newtonian Fluids in T-Junction Parallelized Microchannels. Chem Eng J 471:144303. 10.1016/j.cej.2023.144303 36724386

[CR29] Wiśniewska Małgorzata, Chibowski Stanisław, Urban T, and Dariusz Sternik (2011) Investigation of the Alumina Properties with Adsorbed Polyvinyl Alcohol. J Therm Anal Calorim 103(1):329–337. 10.1007/s10973-010-1040-1

[CR30] Zhao B, Sivasankar VS, Subudhi SK, Sinha S, Dasgupta A, and Siddhartha Das (2022) Applications, Fluid Mechanics, and Colloidal Science of Carbon-Nanotube-Based 3D Printable Inks. Nanoscale 14(40):14858–14894. 10.1039/D1NR04912G 36196967

